# Multi-omics data integration for the identification of biomarkers for bull fertility

**DOI:** 10.1371/journal.pone.0298623

**Published:** 2024-02-23

**Authors:** Valentin Costes, Eli Sellem, Sylvain Marthey, Chris Hoze, Aurélie Bonnet, Laurent Schibler, Hélène Kiefer, Florence Jaffrezic

**Affiliations:** 1 Université Paris-Saclay, UVSQ, INRAE, BREED, Jouy-en-Josas, France; 2 Ecole Nationale Vétérinaire d’Alfort, BREED, Maisons-Alfort, France; 3 R&D Department, ELIANCE, 149 rue de Bercy, Paris, France; 4 Université Paris-Saclay, AgroParisTech, INRAE, GABI, Jouy-en-Josas, France; 5 INRAE, MaIAGE, Université Paris-Saclay, Jouy-en-Josas, France; Universidade Federal do Parana, BRAZIL

## Abstract

Bull fertility is an important economic trait, and the use of subfertile semen for artificial insemination decreases the global efficiency of the breeding sector. Although the analysis of semen functional parameters can help to identify infertile bulls, no tools are currently available to enable precise predictions and prevent the commercialization of subfertile semen. Because male fertility is a multifactorial phenotype that is dependent on genetic, epigenetic, physiological and environmental factors, we hypothesized that an integrative analysis might help to refine our knowledge and understanding of bull fertility. We combined -omics data (genotypes, sperm DNA methylation at CpGs and sperm small non-coding RNAs) and semen parameters measured on a large cohort of 98 Montbéliarde bulls with contrasting fertility levels. Multiple Factor Analysis was conducted to study the links between the datasets and fertility. Four methodologies were then considered to identify the features linked to bull fertility variation: Logistic Lasso, Random Forest, Gradient Boosting and Neural Networks. Finally, the features selected by these methods were annotated in terms of genes, to conduct functional enrichment analyses. The less relevant features in -omics data were filtered out, and MFA was run on the remaining 12,006 features, including the 11 semen parameters and a balanced proportion of each type of–omics data. The results showed that unlike the semen parameters studied the–omics datasets were related to fertility. Biomarkers related to bull fertility were selected using the four methodologies mentioned above. The most contributory CpGs, SNPs and miRNAs targeted genes were all found to be involved in development. Interestingly, fragments derived from ribosomal RNAs were overrepresented among the selected features, suggesting roles in male fertility. These markers could be used in the future to identify subfertile bulls in order to increase the global efficiency of the breeding sector.

## Background

Male fertility is an important economic trait in the cattle industry and more specifically in the dairy sector where a high proportion of animals are bred by artificial insemination (AI) using semen from high genetic merit bulls. The use of subfertile bulls causes economic losses for different actors in the breeding industry, from the breeders that purchase semen to the breeding companies responsible for its production and commercialization. Using semen with reduced fertility also delays calving and lactation and requires multiple interventions by artificial insemination technicians, thus decreasing the global efficiency and sustainability of the dairy sector [1]. For these reasons, identifying subfertile bulls and limiting the dissemination of subfertile semen to herds are important challenges for the breeding sector.

During the past decade, numerous studies have investigated the prediction of bull fertility based on genotypes or semen functional parameters. However, the levels of predictive accuracy achieved during these studies were insufficient [2–7]. As well as genetics and semen parameters, a broad range of epigenetic mechanisms such as DNA methylation, sncRNAs and the ratio between histones and protamines also contribute to male fertility [7–11]. For this reason, epigenetic studies have been the subject of increasing attention in recent years in the context of male fertility, and encouraging results have been obtained [12–19]. However, DNA methylation appears to be insufficient to explain the entire variance related to bull fertility [18]. Male fertility is a complex and multifactorial phenotype, and considering only one type of biological data at a time may indeed not suffice to achieve good levels of prediction, as many contributing factors are not taken into account. To our knowledge, however, studies to explore the potential of several types of epigenetic mechanisms to predict male fertility, in combination with genotypes and semen parameters, have not so far been published.

In the past few years, multi-omics integration analyses have gained popularity among researchers thanks to the development of affordable high-throughput technologies enabling the collection of different types of data from the same biological samples. In order to analyse these data and create interpretable representations, a large number of statistical techniques have emerged in the field of data integration, which continues to be an active research area [20–23]. These include approaches to explore the data and perform descriptive analyses, and notably Multiple Factor Analysis (MFA) that can help to study the relationships between different datasets and detect the principal sources of variation among them, in order to explore the factors causing this variation [24]. Another important topic addressed in multi-omic data analyses is the identification of biomarkers to predict a specific phenotype, the most popular methods being Machine Learning tools such as Random Forest, Gradient Boosting and Neural Networks [25–28].

Despite the abundance of techniques available for data integration, no integrative studies in the field of male fertility have been published to date, probably due to the need for large cohorts and related budget issues, as well as the expertise required to generate and analyse different types of data. During the present study, we integrated genetic (SNPs), epigenetic (DNA methylation at CpGs, sncRNAs) and physiological (semen functional parameters) datasets obtained on a unique cohort of 98 bulls, which is quite large in the context of -omics analyses. We report here on the links between these datasets and bull fertility that were explored using MFA. We also built models from these different biological features using four machine learning methods before assessing their performance and investigating the biological functions of the features selected. To our knowledge, this study represents the first attempt to integrate different types of biological data in the field of bull fertility.

## Materials and methods

### Preparation of semen samples

Ethics statement: not applicable since only commercial samples were used for the purpose of this study.

The animal cohort included 98 French bulls of the Montbéliarde breed that had been classified as fertile or subfertile based on a corrected non return rate (NRR) at 56 days (S1 Fig in S1 File). These 98 bulls were commercialized by two different breeding companies: Evajura (n = 43, maintained at Lons-le-Saunier, France) and Umotest (n = 55, maintained at Brindas, France), and 8 to 10 cryopreserved commercial semen straws were pooled from all of them to constitute the biological sample which was used to extract genomic DNA and total RNAs from respectively 20 and 40 million sperm cells, and to measure semen functional parameters.

### Data Acquisition

The semen functional parameters were obtained in a previous study conducted on the same samples (Costes et al, 2022) and are related to motility (percentage of motile sperm, progressivity, average path velocity, straightness, straight line velocity and amplitude of lateral head), mitochondrial potential and viability. They were assessed using computer assisted sperm analysis (CASA IVOS II, Hamilton Thorne, IMV Technologies) and flow cytometry (Guava). The different protocols used to assess the semen functional parameters, and the different phenotypes are precisely described elsewhere [18]. DNA methylation was investigated using reduced representation bisulphite sequencing (RRBS) and the analysed CpGs that co-localized with variants listed in the “1000 Bull Genomes” database were filtered out. These steps are precisely described in [18]. RNA extraction, sncRNA library preparation and bioinformatics analysis were performed as described elsewhere [8]. Further, the sncRNA counts were normalized according to library depth using the R package DESeq2 [29]. Genotypes obtained using the EuroGMD 50K DNA chip (Illumina) were supplied by the breeding companies and represented 40,479 SNPs that successfully passed quality controls. Consequently, each bull was described by 2,003,005 features that belonged to four different biological datasets (40,479 SNPs, 1,548,563 CpGs, 413,952 sncRNAs and 11 semen parameters). A description of each sample, together with the semen parameters measured, is given in the following table (S1 Table).

### Data integration by multiple factor analysis

To achieve an integrated analysis of these data, we first applied an exploratory approach, namely the Multiple Factor Analysis (MFA). This statistical technique consists in establishing a linear combination of features in order to maximise the variance of the table analysed in different principal components, as in Principal Component Analysis (PCA). The difference is that MFA is able to deal with multi-type data that may either be quantitative or categorical. Furthermore, each value within a dataset is standardized by the first singular value of this dataset, in order to render the different sets of data comparable whatever their dimensions. The FactoMineR (v2.4) package was used for MFA analyses, and the graphics were obtained using the factoextra (v1.0.7) package [30]. DNA methylation, sncRNA and semen parameter data were encoded as numerical features and the SNPs were encoded as categorical features, thus precluding their projection on the same variable factor map as the aforementioned numerical features. Fertility, extraction batch and semen collection centres were considered as supplementary categorical features so they did not contribute to the MFA construction. All numerical features were scaled prior to the analysis in order to permit multi-table comparisons.

Random Forest, Gradient Boosting, Lasso Logistic regression and Neural Networks were used to build predictive models and perform feature selection. A brief description of each method is presented below, as well as the parameters used for each analysis.

### Logistic Lasso

The regularised Logistic Lasso was used as the feature of interest is categorical with two modalities, namely “Fertile” / “Subfertile”, and can be explained by a large quantity of features. The logistic regression is expressed by the equation below to calculate the probability of an individual of being in class 1, depending on different *x*_*i*_ values.


$$P(1|X)=\frac{e^{b_{0}+b_{1}x_{1}+\cdots +b_{j}x_{j}}}{1+e^{b_{0}+b_{1}x_{1}+\cdots +b_{j}x_{j}}}$$

As in the case of linear regression problems, the purpose is to estimate the different coefficients of the model (*b*_*j*_). This is achieved by determining the coefficients that maximise the likelihood. In this study, as in most -omics analyses, the number of features was much larger than the number of observations (n = 98). We therefore applied Lasso regularisation, and the coefficients of the model were estimated by maximising the likelihood under an L1 penalty [31]. Two hyper parameters were tuned: lambda (coefficient of regularisation) with the following grid [0.01, 0.1, 1, 5, 7, 10] and epsilon (tolerance termination criterion) [0.0005, 0.005, 0.001, 0.05, 0.1, 0.15, 0.2]. The tuning was done by a 10 fold cross validation, and the chosen values were the ones maximising the AUC (Area Under the ROC Curve) on the testing fold. Using this procedure, the lambda parameter was set to 7 and epsilon to 0.5.

### Random forest

Random Forest is an ensemble method based on classification trees which in this case were applied using the h2o R package (v3.32.1.3) with the “h2o.randomForest” function and the party R package (v1.3.7) with the “Cforest” function. This technique constructs K trees for K subsets of individuals and features. Subsets are obtained by bo otstrapping the individuals and sampling a number (mtry) of random features in the original dataset for each node calculation. During this study, K was set at 500 and the mtry parameter was calculated as the square root of the number of features in the original dataset. In each subset, trees were constructed recursively from the original node until the last one by splitting the parent node into two child nodes, using the features that best discriminated between fertile and subfertile bulls. A tree stops growing if a node fulfils one or more of these three conditions: (i) the leaf contains only one individual; (ii) the maximum depth has been reached (in the present case, 20); (iii) the split of the node does not sufficiently improve the classification (in a node, the squared error reduction must be greater than 0.00001). The importance of features in Random Forest was calculated here by looking at the reduction in the squared error before and after a split node. The reduction was attributed to the features responsible for the split. These reductions were then summed for each feature in each tree, thus indicating which features were the most relevant (the higher the value, the more relevant the feature). During this study, both classic and Cforest versions of the Random Forest approach were applied, with respectively the CART and Ctree procedures being used to build the classification trees [32,33]. Briefly, the CART procedure selects the features that minimise the Gini impurity criterion, while Ctree first identifies an explanatory feature that is correlated with the variable of interest before choosing the best split within this feature that minimises the Gini impurity criterion.

### Gradient boosting

Gradient boosting is also an ensemble method based on classification trees, but the strategy used to build the trees differs from that used by Random Forest; unlike Random Forest where each tree is constructed independently from the others, a tree (n+1) is built from the “error”(the residual) of tree (n) in gradient boosting. Further details on the precise algorithm for gradient boosting can be found in Hastie et al. [34]. Three parameters were tuned in this method: the learning rate with the following grid [0.1, 0.2, 0.3, 0.4, 0.5], the maximum depth [1, 3, 5, 7, 9, 11] of a tree and the number of trees [500, 1000, 1500]. The tuning was performed using one training and one testing set, and by choosing the values maximising the AUC on the testing set. No cross-validation was done in this case because it included more parameters to be assessed and required an extensive computing time. Using this procedure, the maximum depth was set to 5, the number of trees was 1500 and the learning rate 0.1. This method was implemented using the h2o package (v3.32.1.3) with the h2o.gbm function.

### Neural network

The Neural Network method is based on a combination of artificial neurons distributed between different layers. An artificial neuron is described using the mathematical formula below, where *x*_*i*_ is the output of neuron i, *w*_*i*_ is the weight given to the output of neuron i, and *φ* is the activation function. The *w*_*o*_ weight is associated with a fictitious feature *x*_*o*_ that is equal to 1.


$$\varphi [\sum_{i=0}^{m}w_{i}x_{i}]$$

Each layer may contain a different number of neurons, connected so that each neuron in layer (n+1) is connected to all neurons in layers n and (n+2). The deep learning network is calibrated to obtain a prediction by estimating the different weights (*w*_*i*_) through a process called gradient backpropagation which is performed after each sample passes through the neural network [35]. Four parameters were tuned: the hidden layers architecture with the following grid ([200,100,50], [100,50,25], [100,50]), the learning rate [0,05,0.1,0.2], the L1 penalty [0,0.1,1,5] and the activation function [Rectifier, Tanh]. The tuning was done using one training and one testing set, and by choosing the values maximising the AUC on the testing set. Using this procedure, the hidden layers architecture was set to [100, 50, 25], the learning rate to 0.1, the L1 penalty to 0.1 and the chosen activation function was the Rectifier. The Neural Network algorithm was applied here using the h2o package (v3.32.1.3), with the h2o.deeplearning function. The calculation of relative feature importance was implemented directly in the package using the Gedeon method [36].

Random Forests were first used to pre-select relevant sncRNA, CpG and SNP features and allow a dimension reduction (see Results). Then, all four different methods were applied to a matrix that contained the DNA methylation values at CpGs, sncRNAs expression, genotypes at SNPs and semen parameters for each individual. In both cases, the methodology was the same: the animal cohort was split into two sets: training and testing. The training set contained 2/3 of the samples (n = 65) selected randomly but retaining the original proportion of fertile and subfertile samples, and was used to create the model. The testing set contained the remaining samples (n = 33, or 1/3 of the cohort) to evaluate the predictive ability of the model previously constructed on the training set. This process was iterated 50 times with resampling of the training and testing sets and the AUC was averaged over the 50 AUC values obtained for each iteration [37].

### Feature annotation and enrichment analysis

The gene annotation of CpGs and SNPs was performed relative to gene features with an in-house pipeline, as described in [18]. The reference files were downloaded from Ensembl (ftp://ftp.ensembl.org/pub; release 95). The following criteria were applied: TSS, -100 to +100 bp relative to the transcription start site (TSS); promoter, -2000 to -100 relative to the TSS; TTS, -100 to +100 relative to the transcription termination site (TTS). Genes containing CpGs and/or SNPs in intragenic, upstream (up to -10 kb from the TTS) or downstream regions (up to +10 kb from the TTS) were subjected to an enrichment analysis using DAVID (version 6.8) with default parameters and using all genes (n = 20,641) targeted by the analysed SNPs and CpGs as a reference.

The gene targets of the miRNAs were identified using TargetScan (version 7.2) with default parameters, and the enrichment analysis was performed on the identified genes using WebGestalt against the whole genome.

## Results

### Data preparation and extraction of relevant features

**Fig 1:**
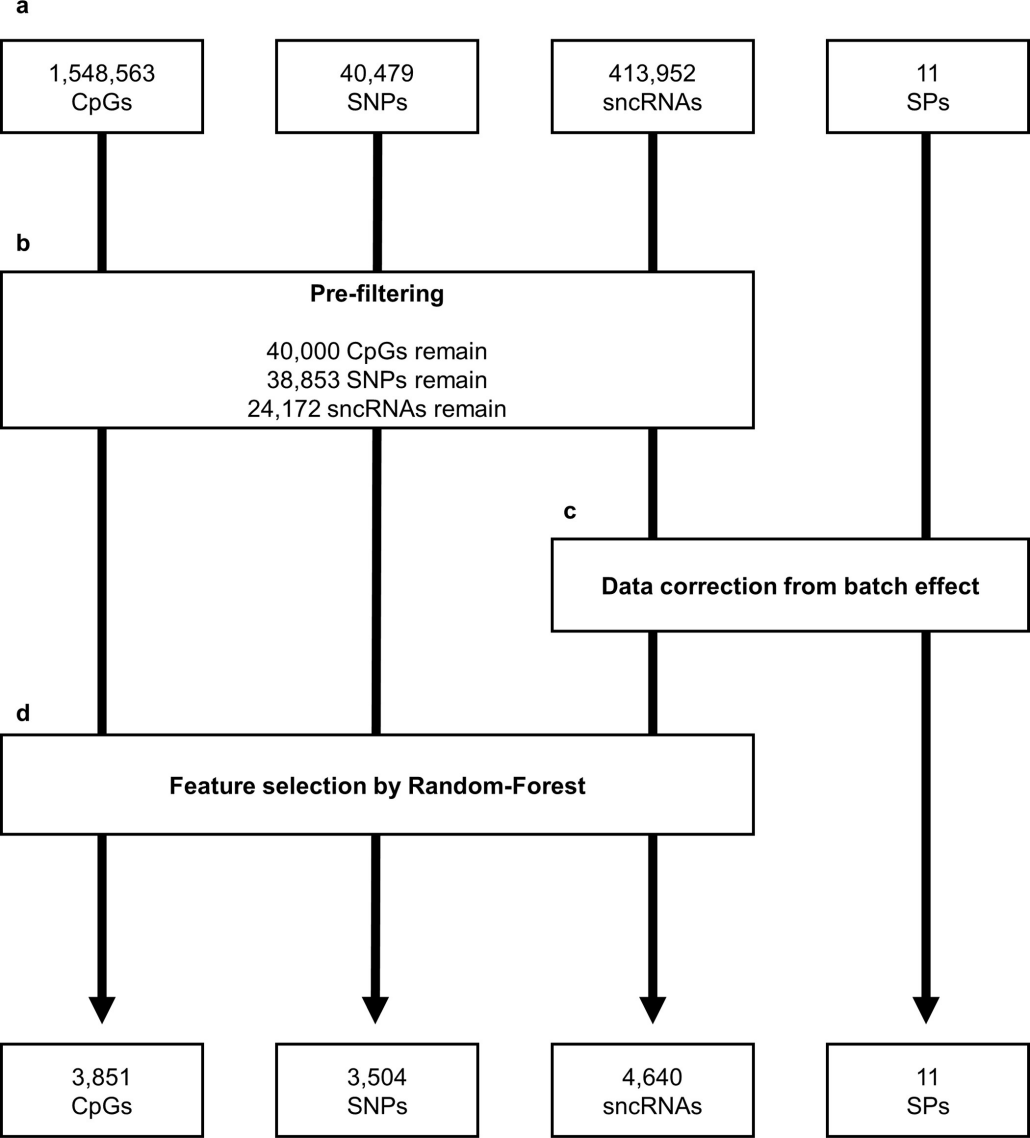
Data filtering strategy. The four different tables included a heterogeneous number of features **[a].** Because the CpGs, SNPs and sncRNAs constituted huge data tables, features that could be considered as noise and features that did not display significant variations among the bulls were filtered out **[b]**. Because the remaining sncRNAs and SPs were impacted by the extraction batch of the semen, they were next corrected for this batch effect **[c]**. Finally, because the CpG, SNP and sncRNA tables still included an important number of features, the most relevant were selected using a supervised method, Random Forest **[d]**. At the end of these three filtering steps, 12,006 relevant features originating from four data tables were retained for further analysis.

In order to achieve data integration and construct a model predictive of bull fertility status, we considered four biological datasets relevant to male fertility: sperm DNA methylation and sncRNAs, semen parameters (SPs), and the genotypes of the bulls. These different tables included 1,548,563 CpGs (DNA methylation features), 413,952 sncRNAs, 11 SPs and 40,479 SNPs (genotype features), respectively (Fig 1A).

The first step in the analysis was to pre-filter the DNA methylation, sncRNA and SNP data tables in order to remove unusable or unnecessary features and limit the dimensions of these tables (Fig 1B). The DNA methylation table included a large number of missing values resulting from CpGs uncovered in some samples, or CpGs covered by less than 10 reads and from which DNA methylation values could not be estimated accurately. As some of the methods we used could not handle missing values, each CpG that contained at least one missing value across the 98 samples was filtered out, which left 641,306 CpGs with no missing values. Assuming that CpGs with extreme methylation values displayed little inter-individual variability, we then removed the CpGs that were consistently hypo- (0% to 20%) or hyper-methylated (80% to 100%) in all the samples; from the 98,203 remaining CpGs, we selected the 40,000 CpGs with the highest variance. The sncRNA table also contained a very large number of features, many of them being expressed below the detection level in most samples. Because these expression traces could not be quantified thoroughly in all samples, the corresponding sncRNAs were disregarded during subsequent analyses. Only sncRNAs that displayed an average read count higher than 10 after normalisation were therefore retained, which represented 24,172 features in total. Finally, SNPs at which the genotype was identical for all 98 bulls were filtered out, resulting in our cohort containing 38,853 SNPs with polymorphism.

Unlike the genotypes that are obtained routinely using standardized procedures, DNA methylation, sncRNA and SP data were acquired in the lab from the 98 semen samples processed in different batches. We had already demonstrated that DNA methylation was not significantly affected by the batch [18], and this was also confirmed in the current study (S2 Fig in S1 File). However, a PCA run on the 24,172 selected sncRNAs and 11 SPs revealed that the batch had a huge impact on these data (S3 Fig in S1 File); this effect was confounded with the origin of the bulls in terms of semen collection centres. We therefore corrected the data for the batch effect using a generalized linear model for the sncRNAs and a linear model for SPs, with the experimental batch as a fixed effect. The residuals of the model were then extracted. The corrected data were no longer biased according to the batch or centre (Fig 1C) (S3 Fig in S1 File), and could therefore be used with confidence for subsequent analyses. This correction for the batch effect did not apply to the SNP data, which are routinely obtained using standardized procedures. However, centres 1 and 2 were distinguished on the first dimension of the PCA (S4 Fig in S1 File), thus indicating that these two centres probably select and commercialize slightly different genetics.

As shown in the following supplementary figures (S2–S4 Figs in S1 File), the largest part of the variance in the 24,172 sncRNA, 11 SP, 40,000 CpG and 38,853 SNP data was unrelated to fertility. The final step in data preparation therefore involved selecting features relevant to male fertility in a supervised manner (i.e., selecting features that could differentiate fertile from subfertile bulls), using a Random Forest approach (Fig 1). Due to the small number of features in the SP dataset, only the DNA methylation, sncRNA and SNP data underwent this step which resulted in the selection of 3,851 CpGs, 3,504 SNPs and 4,640 sncRNAs. Selection was based on the relative importance of each feature in the model (S5 Fig in S1 File).

At the end of these pre-processing steps, a table containing 12,006 features originating from four different biological datasets, with relatively balanced proportions (except for SPs) was therefore obtained and used during subsequent analyses. These features are shown in the following supplementary table (S2–S5 Tables).

### Integration by multiple factor analysis

We next investigated the potential links between the different types of data and their relationships with male fertility through MFA of the four datasets shown in Fig 1D.

**Fig 2:**
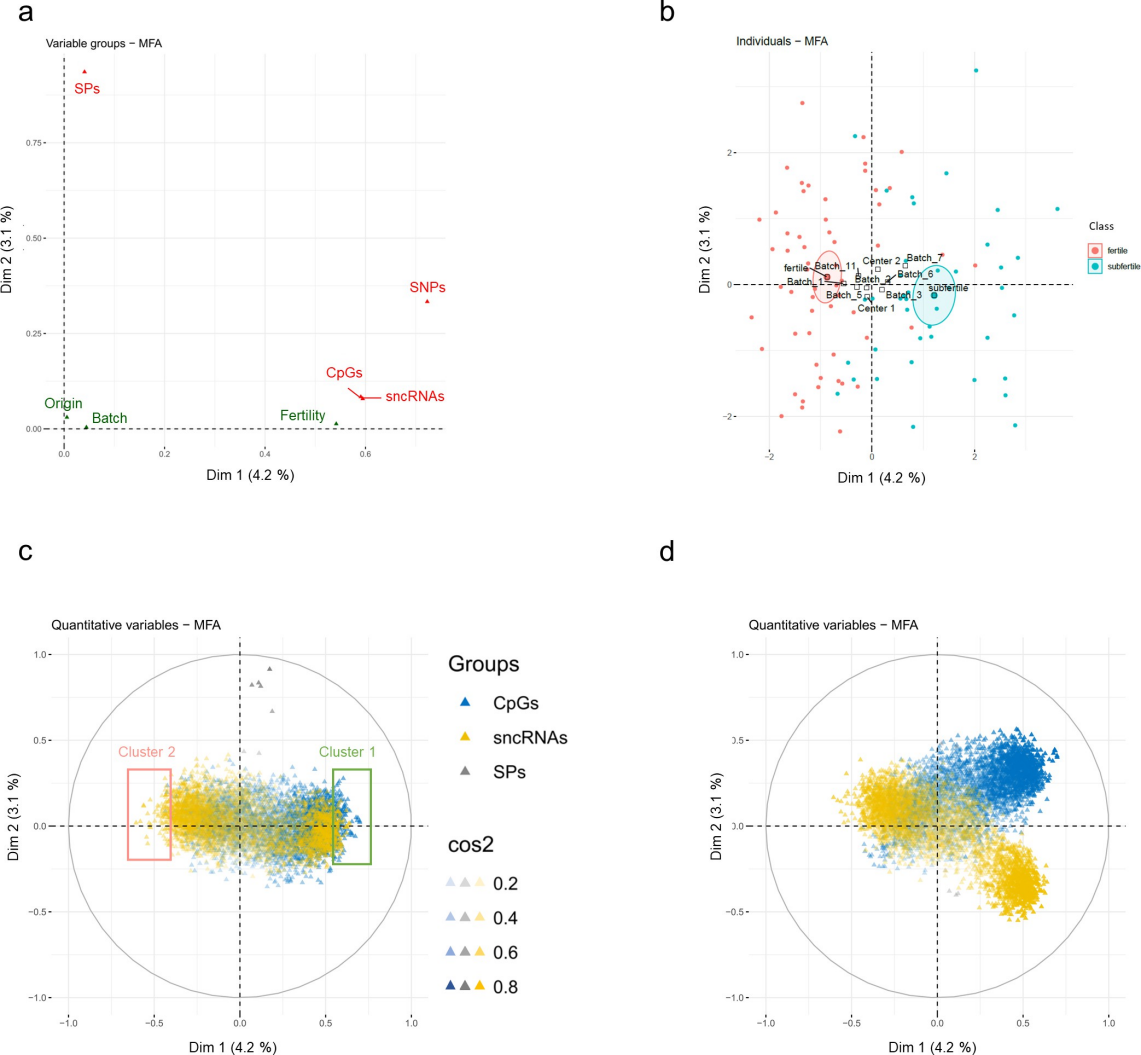
Multiple factor analysis highlights the contributions of SNPs, CpGs and sncRNAs to bull fertility. MFA was run on the 12,006 selected features belonging to the CpG, sncRNA, SNP and SP tables that actively contributed to the results. Furthermore, fertility, the origins of bulls and the semen extraction batch were set as illustrative features, meaning that they did not participate in MFA construction. **a:** A global variable plot with active features shown in red and illustrative features in green. **b:** Individual factor map where each dot corresponds to a bull and coloured depending on its fertility class. C, D: Variable factor maps for quantitative features (CpGs and sncRNAs). The first and second dimensions [c] and the first and third dimensions [d] are represented. Each arrowhead corresponds to a feature and was coloured depending on its dataset of origin, with CpGs, sncRNAs and SPs shown in blue, yellow and grey, respectively. Furthermore, the intensity of the colour of arrowheads indicated the cos^2^, reflecting the strength of the correlation between a feature and dimension 1. In C, two clusters are represented, gathering the features with the most important positive (>0.55, cluster 1) or negative (<0.4, cluster 2) coordinates along dimension 1.

The global factor map (Fig 2A) shows which types of features contributed to dimensions 1 and 2 among SPs, sncRNAs, CpGs and SNPs, and indicates the positioning of supplementary variables (fertility, semen processing batch, origin of the bulls according to semen collection centre). The CpG, sncRNA and SNP datasets all exhibited high coordinates on the first dimension, meaning that they represented the most important sources of variation and were the main contributors to the first MFA component. However, SPs contributed little to the first dimension but much to the second. Fertility exhibited a high coordinate on the first dimension and a very low coordinate on the second, meaning that the most important sources of variation in the CpG, sncRNA and SNP datasets were correlated to the fertility status of the bulls. This result was expected given that MFA was conducted on features preselected on their ability to discriminate fertile and subfertile bulls. On the other hand, fertility was not associated with the second dimension, suggesting that in the studied cohort the fertility status of the animals was independent from the SPs. Finally, and consistent with the correction of the data for these effects, bull origin and batch did not contribute significantly to the two first dimensions of MFA. As expected given the correlation of fertility with the first dimension, fertile and subfertile bulls were discriminated along this dimension (Fig 2B).

### Correlation structure among CpG and sncRNA features

Because quantitative and qualitative features cannot be displayed on the same MFA variable factor map, SNPs were not considered for the correlation analysis. In line with the above observations, features belonging to the CpG and sncRNA datasets contributed to the first MFA dimension while SPs contributed to the second (Fig 2C). Both features with positive and negative coordinates on the first dimension of the factor map were found, indicating positive and negative correlations with fertility status. To identify the most relevant features, two clusters were defined based on the most extreme coordinates on the first dimension. Cluster 1 contained the CpGs and sncRNAs (170 CpGs and 83 sncRNAs) that were more methylated or expressed in subfertile bulls than in fertile bulls (subfertile bulls having positive coordinates on the individual factor map; Fig 2B) and cluster 2 mostly contained sncRNAs (120 sncRNAs and only three CpGs) that were more expressed in fertile bulls than in subfertile bulls. Interestingly, most of the scnRNAs belonging to these clusters were sncRNAs of the miRNA and rRF (ribosomal RNAs derived fragment) families (S6 Fig in S1 File).

**Fig 3:**
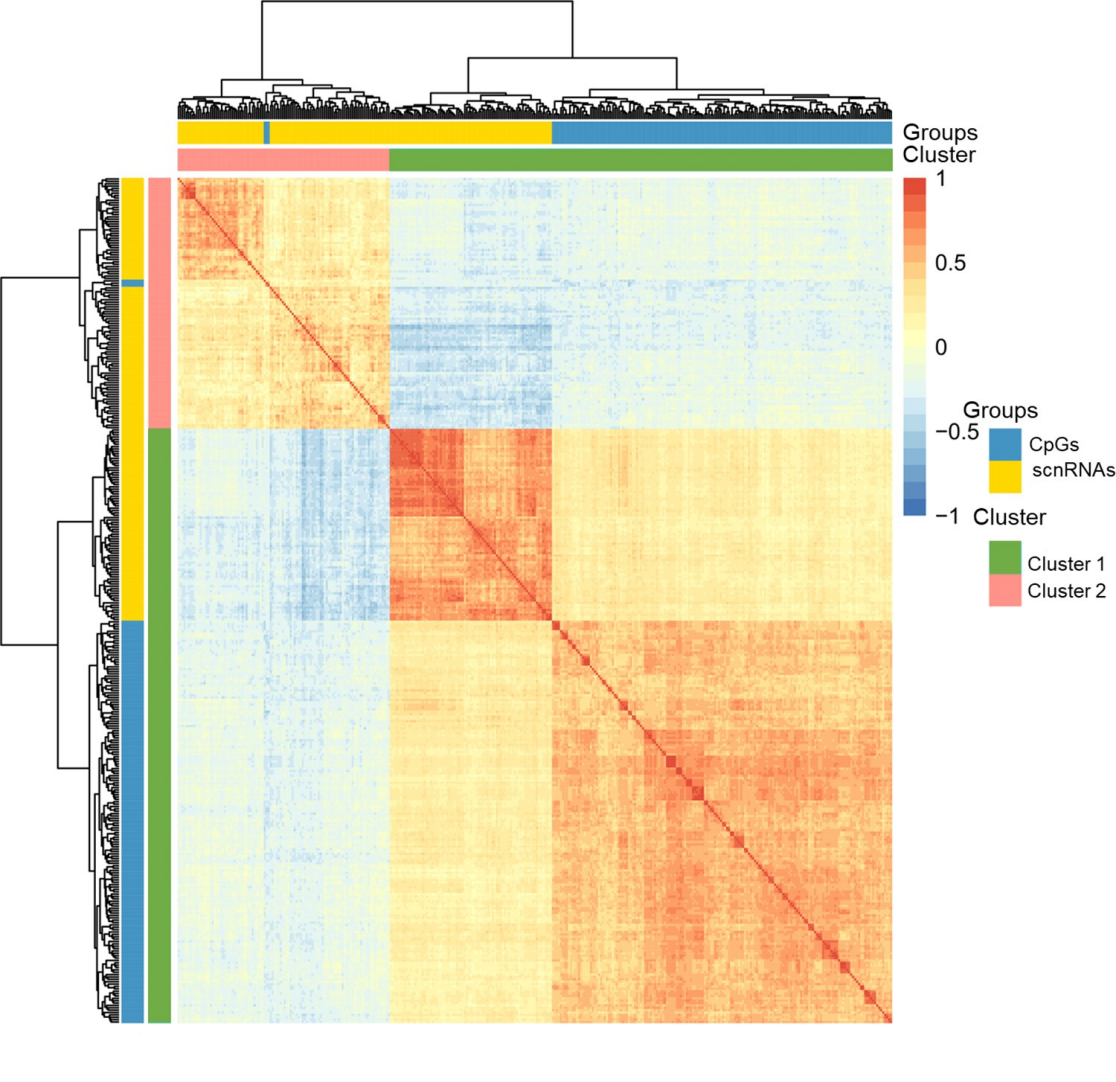
Correlation structure among CpG and sncRNA features. The correlation matrix for features belonging to the two clusters defined in Fig 2C was computed. Features are displayed in lines and columns and coloured according to the datasets and clusters. The intensity of colours in the heatmap reflects the strength of the correlation between two features, with positive and negative correlations indicated in red and blue, respectively.

The potential correlation structure among features belonging to the CpG and sncRNA datasets was then investigated, with a particular focus on cluster 1 where CpGs and sncRNAs were represented in equivalent proportions. By looking at the feature graph on Fig 2C it can be seen that the arrowheads from CpGs and scnRNAs were close together, suggesting a correlation structure among these two types of biological features. However, these features were not highly correlated with the first dimension (cos^2^ with the first dimension was not high), and the arrowheads could be distant from each other on other dimensions, as illustrated using the first and third dimensions where the orthogonal structure between sncRNAs and CpGs suggested very limited correlations (Fig 2D). This was further confirmed by the correlation heatmap (Fig 3) revealing an absence of correlation structure between the CpGs and scnRNAs, while positive correlations were observed within each dataset of cluster 1.

To summarise this part, the MFA results demonstrated that genotypes, sperm sncRNA expression and DNA methylation all contributed separately to fertility, with limited correlations between features of the sncRNA and CpG datasets.

### Comparison of several methods for feature selection

After calibration of the different parameters, the prediction models were applied to the four datasets. Here the goal was to use the different predictive models in order to identify the features that are the most important for the prediction. The predictive ability of the model was used as a criterion to select the most informative features but not analysed *per se*, due to the absence of an independent cohort on which it could be accurately assessed (see Discussion).

**Fig 4:**
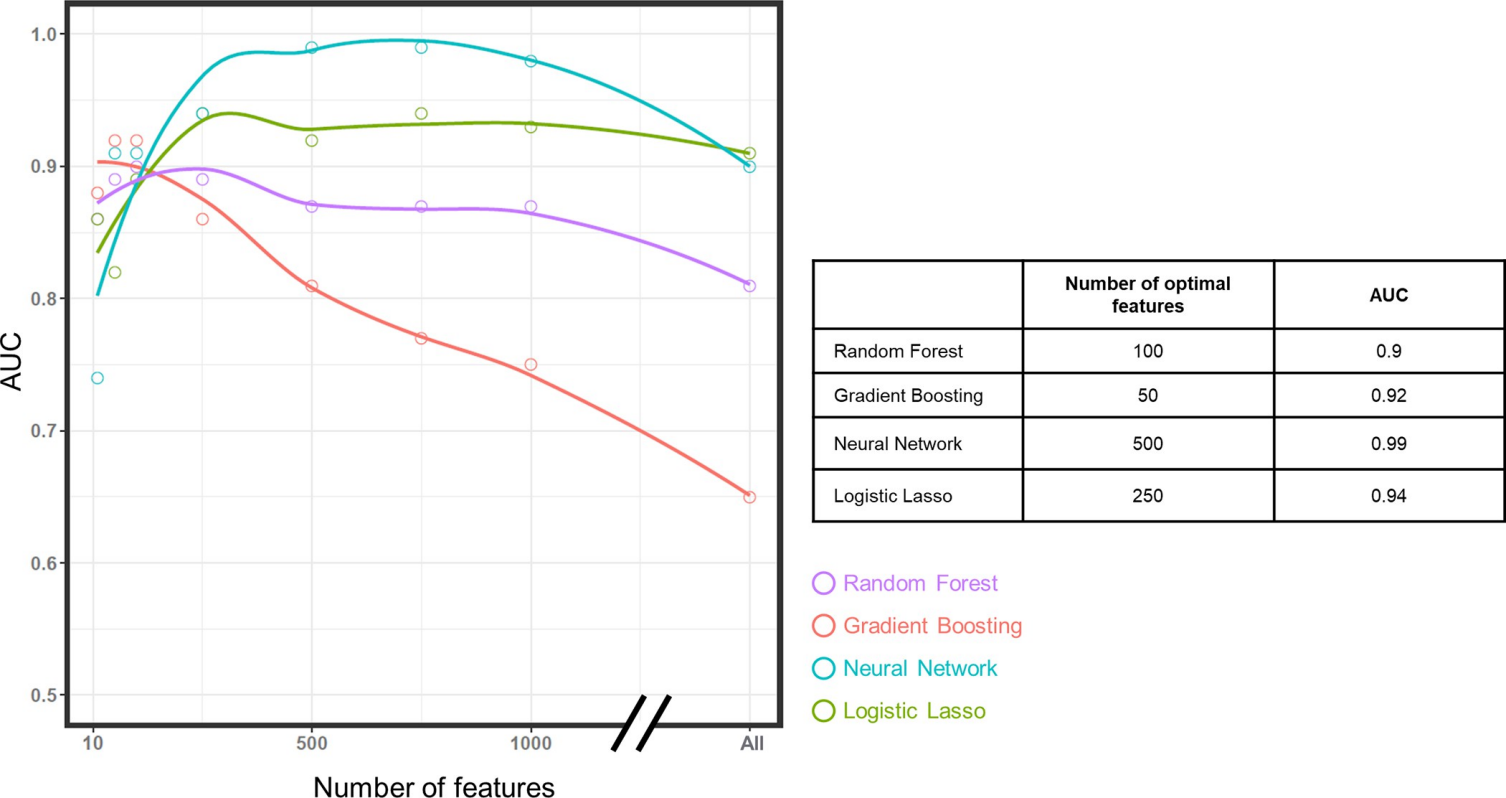
Predictive accuracy of bull fertility and optimal number of features for the different methods. For each method, one model was constructed with the 12,006 features, these being classified depending on their importance. Then, for each method, models were constructed with the top 1000, 750, 500, 250, 100, 50 and 10 features. Using this information, the figure on the left-hand side indicates the AUC on the y-axis and the number of features used during model construction on the x-axis. Each dot (coloured according to the method) represents the actual AUC values obtained for each model. A tendency curve was also drawn for each method using the geom_smooth function of the ggplot2 package with default parameters. The table on the right-hand side shows the optimal number of features and the associated AUC values obtained for each method, based on the actual AUC values and not on the tendency curve.

For each method, a model was first constructed using the 12,006 features. The number of features included in the model was then gradually decreased, and an AUC value was calculated in each case (Fig 4, left panel). Interestingly, each method exhibited a specific behaviour regarding the optimal number of features. For example, Neural Networks displayed poor performance with small numbers of features (AUC = 0.74 with 10 features), but greatly improved when including all the features in the model (AUC = 0.9). By contrast, Gradient Boosting outperformed with a small number of features in the model (AUC = 0.88 with 10 features) but was not robust with a large number of features (AUC = 0.65 with all features). Most importantly, and despite these different behaviours, all methods correctly identified features that bring useful information for the prediction, as illustrated by the minimal number of features that maximised the AUC for each method (Fig 4, right panel). For further analysis and for each method, the features that maximise the predictive performance were kept which correspond to 100 features in Random Forest (AUC of 0.9), 50 in gradient boosting (AUC of 0.92), 250 in Logistic Lasso (AUC of 0.94) and 500 in Neural Network (AUC of 0.99).

### Type of selected features

To gain insight into the information crucial for fertility prediction that was embedded in the four datasets, we studied the features selected by the four methods in more detail, paying a particular attention to their biological nature.

**Fig 5:**
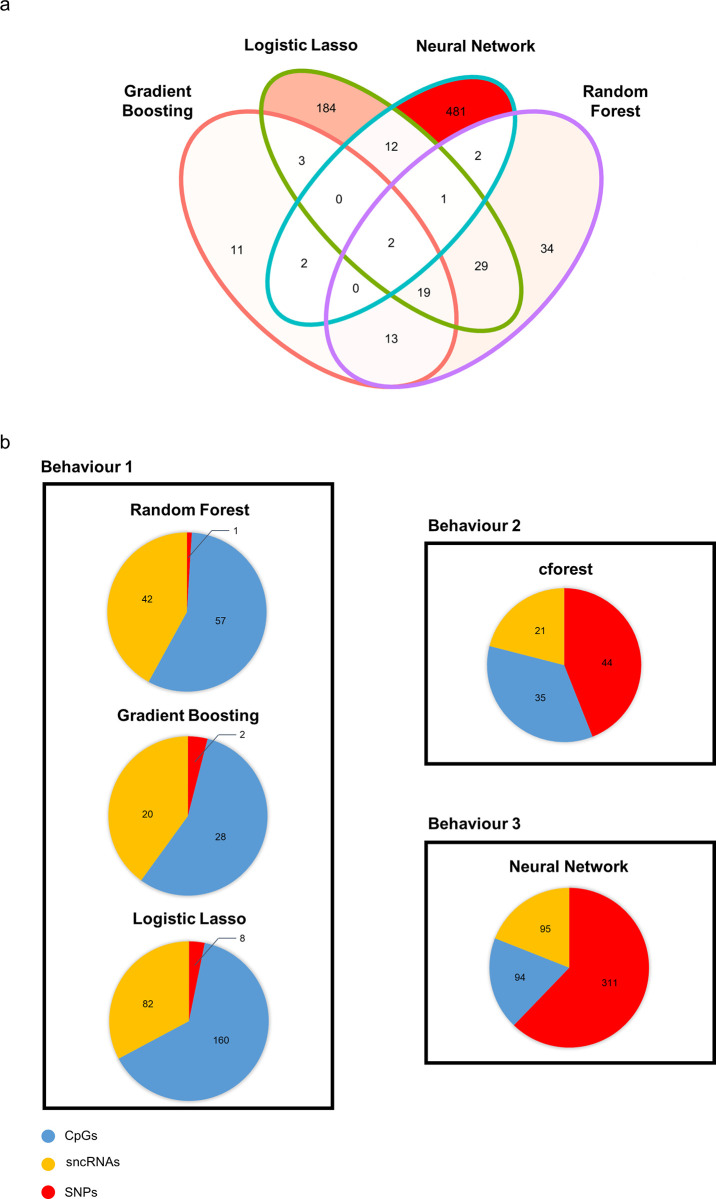
The features selected are specific to each method. **a:** Venn diagram showing the intersection between methods in terms of the features selected. Areas are coloured according to the proportion of features they include, compared to the total quantity of features selected by each method. **b:** The datasets of origin of the selected features are represented by pie charts. Methods displaying similar behaviours are grouped together.

For this, we first of all compared the optimal features selected by the four methods (Fig 5A). Strikingly, only two features were common to all four methods, demonstrating the specificity of each method, although there was some overlap. Indeed, 66% of the features selected by Random Forest were also selected by at least one other method, and this percentage reached 88% for Gradient Boosting. In contrast, the methods using larger numbers of features did not share many features with other models (26% for Logistic Lasso and only 3% for Neural Network).

The nature of the selected features was then analysed for each method (Fig 5B). Whichever method was employed, SPs were never identified as important features for fertility prediction, a finding in line with the absence of correlation between SPs and bull fertility observed during MFA. Logistic Lasso, Random Forest and Gradient Boosting behaved quite similarly, as they mainly selected CpGs and sncRNAs (Fig 5B, left panel). It should be noted that the CART procedure used to construct the classification trees for Random Forest and Gradient Boosting is known to be biased toward the selection of continuous rather than qualitative features [32]. Given the distribution of the–omics features (continuous for CpGs and sncRNAs vs. discrete for SNPs), this property of CART might explain why very few SNPs were selected. However, it should be noted that this selection bias had never previously been reported for Logistic Lasso, which also favoured the selection of CpGs and sncRNAs over SNPs. Interestingly, the Cforest method, which is related to Random Forest but uses the Ctree procedure rather than CART to build the classification trees, was shown to be unbiased toward the data type and distribution [32]. This method therefore offered an opportunity to assess the effect of CART on the nature of the selected features, at least for Random Forest, and was applied to the 12,006 features described above. The number of features that maximised the model performance was 100, similar to the standard Random Forest approach, but predictive accuracy was slightly better (AUC of 0.92 compared to 0.90; (S7 Fig in S1 File)). The pattern of the selected features differed from the classic Random Forest approach, with a much higher proportion of selected SNPs (Fig 5B, upper right panel), thus confirming that CART biased feature selection. Finally, the Neural Network approach displayed another pattern of selection, where SNPs represented the main type of features selected (Fig 5B, lower right panel).

In conclusion, although each method behaved specifically in terms of the number and nature of the features selected, they all selected at least two types of features, thus highlighting the benefits of data integration for the prediction of bull fertility. In addition, all types of–omics features were selected, suggesting that they were all relevant to predicting bull fertility.

### Functional annotation of selected features

In order to determine whether the features contributing to fertility prediction were biologically relevant, we annotated them regarding genes and sncRNA families, with particular focus on the features selected using unbiased methods (Logistic Lasso, Cforest and Neural Networks).

**Fig 6:**
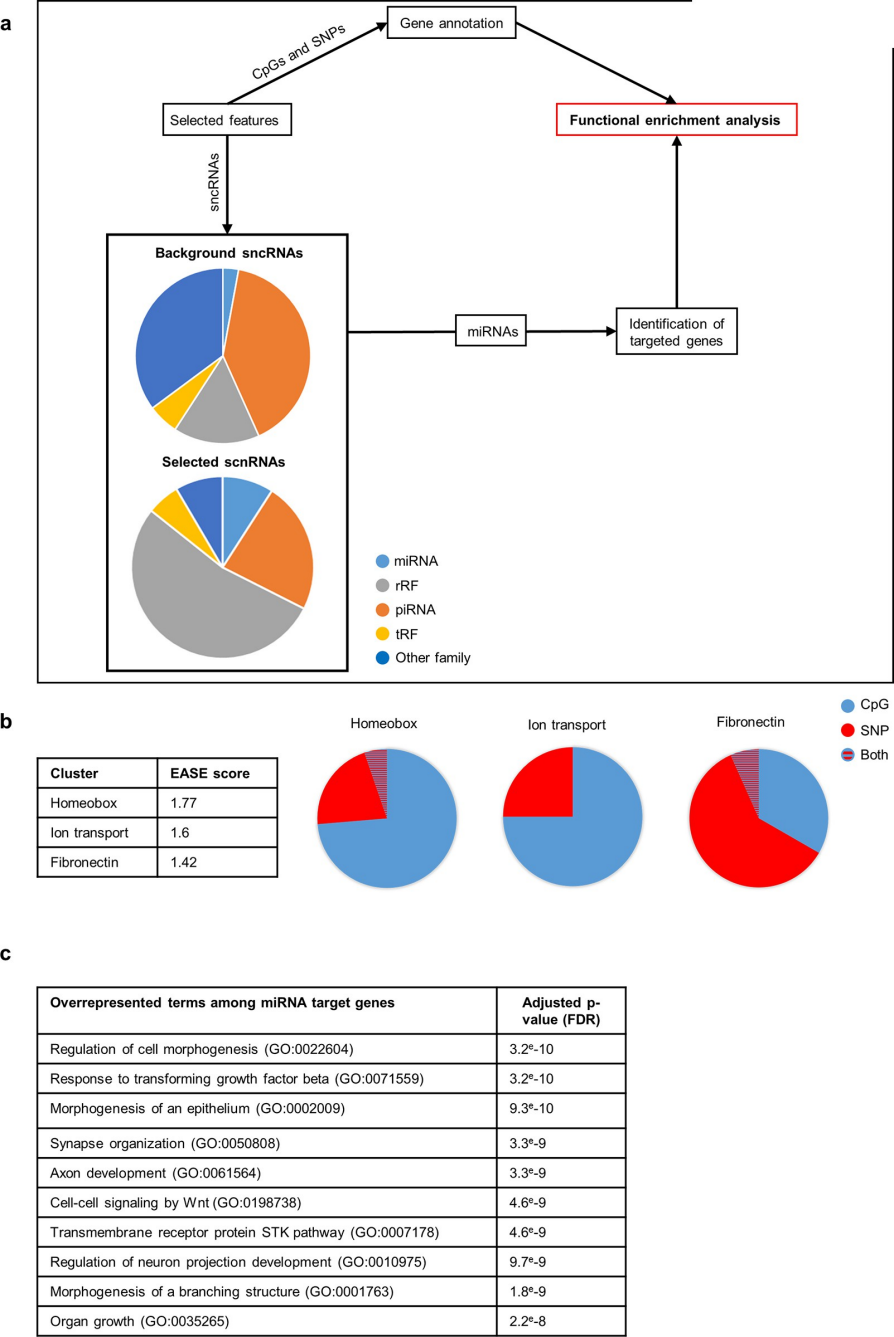
Functional analyses of selected features. **a**: Global strategy for functional analysis. The combination of SNP, CpG and sncRNA features selected by the three unbiased methods (Cforest, Gradient Boosting, Neural Networks) was considered and referred to as “Selected features”. Genes including the selected CpGs and SNPs were subjected directly to enrichment analysis. The distribution of different sncRNAs families highlighted an overrepresentation of miRNAs and rRFs among the selected features when compared to the background, which included the 413,952 sncRNAs that were initially represented in the sncRNA dataset (lower left panel). The analysis therefore focused on the miRNA target genes that were subjected to functional enrichment analysis. **b**: The genes containing selected SNP and CpG features underwent enrichment analysis using DAVID. Three clusters of terms were significantly enriched (EASE score higher than 1.3; left-hand panel). The proportions of genes targeted by selected CpGs only, selected SNPs only, or by both CpGs and SNPs, varied in the three clusters (pie charts, right-hand panel). **c**: Genes identified as putative targets of selected miRNAs by Targetscan underwent an overrepresentation analysis using Webgestalt. The top 10 overrepresented GO terms are listed, with the corresponding adjusted p-values.

We used different strategies depending on the nature of the selected features (Fig 6A). CpG and SNP features were analysed together since they both have the potential to target coding genes directly, thus affecting regulation of their expression. Therefore, the genes containing selected CpGs or SNPs in their body or flanking regions were first identified and then subjected to functional enrichment analysis. The situation was more complex for sncRNAs whose functions vary depending on the family to which they belong [8]. Because tools allowing the *in silico* identification of putative miRNA targets are available, we sought to determine genes potentially targeted by the selected miRNA features and then subjected them to functional enrichment analysis [38].

When each method was analysed separately, no individual gene ontology (GO) term was found to be significantly enriched among genes containing CpG and SNP features selected by any method. One and two clusters with significant enrichment scores (EASE scores above 1.3) were however found using DAVID from the CpG and SNP features selected by Neural Networks (164 gene IDs, (S8 Fig in S1 File)) and Logistic Lasso (64 gene IDs, (S8 Fig in S1 File)), respectively. By contrast, no enriched cluster could be found among the features selected by Cforest that covered only 31 gene IDs. The enriched clusters found with Neural Networks and Logistic Lasso displayed no overlap regarding either genes or terms. Similarly, the distributions of sncRNA families among the selected sncRNA features differed markedly between the three methods (S9 Fig in S1 File), demonstrating the specific behaviour of each method regarding the types and functions of the selected features.

We next grouped the information gained using the three unbiased methods in order to increase the number of targeted genes and hence the relevancy of the functional enrichment analysis. This produced a list of 777 unique features (S5 Table). CpG and SNP features could be associated with 319 gene IDs that did not display any significant enrichment regarding individual GO terms. Three clusters with a significant enrichment score were however found using DAVID; they respectively gathered homeobox-containing genes, genes involved in ionic transport and proteins containing a Fibronectin domain (Fig 6B). The homeobox cluster was of particular interest because genes in this family play key roles in embryonic and foetal development [39]. The fibronectin cluster was also compelling since fibronectin is a glycoprotein important to spermatozoa physiology and plays a role in interactions between gametes [40]. It is worth noting that the CpG features mainly targeted the homeobox and ionic transport clusters, whereas SNPs were mainly annotated to the fibronectin cluster.

We also compared the distributions of the different sncRNA families among the selected features and the background. rRFs and miRNAs were enriched among the selected features, while PIWI-associated RNAs (piRNAs) were depleted and tRFs (transfer RNAs-derived fragments) remained unchanged (Fig 6A). A total of twelve miRNA features were selected by the three methods. Their putative target genes (5601 gene IDs) were identified *in silico* using TargetScan, based on homologies with the miRNA seeds [38]. Enrichment analysis was then performed on these putative targets using WebGestalt (Fig 6C). This method was chosen because DAVID cannot process lists containing more than 3000 genes. GO terms related to cell differentiation, embryonic and foetal development were overrepresented, such as: “Regulation of cell morphogenesis”, “Morphogenesis of an epithelium”, “Synapse organization”, “Axon development”, “Regulation of neuron projection development”, “Morphogenesis of a branching structure” and “Organ growth”. These results therefore suggest that the selected miRNA features potentially regulate genes involved in important developmental processes.

In conclusion, the most predictive features selected by the three methods were annotated to genes and sncRNA families potentially important to development. Changes to their status in subfertile bulls may therefore exert subtle effects on gene expression after fertilisation, resulting in altered developmental outcomes with an indirect negative impact on male fertility.

## Discussion

The purpose of this study was to use data integration methodologies to analyse the links between semen parameters, DNA methylation, sncRNA expression, genetic polymorphism and male fertility in 98 AI bulls with contrasting fertility levels. MFA was run on a total of 12,006 features obtained after data filtering, processing and a first round of feature selection, and highlighted CpGs, sncRNAs and SNPs as the main sources of variability correlated to fertility status. Different methodologies were then used to construct predictive models in order to select features that were the most linked to bull fertility. These features were analysed and interestingly, were at least represented by two types of–omics data and related to male fertility as well as development.

### DNA methylation and sncRNA expression contribute independently to bull fertility

The first interesting finding was that bull fertility was not linked to the semen parameters analysed during this study, which were related to the viability, motility and mitochondrial status of spermatozoa [18]. As shown in our previous research, a high proportion of sperm in each sample was viable post-thawing, with correct motility and sufficient energy supplied by the mitochondria to sustain its function. The absence of any major changes to semen parameters related to subfertility underscores the importance of the research efforts being made in both livestock species and humans to identify potential causes of idiopathic subfertility and infertility with a normozoospermic profile [10,41]. By contrast, features among the CpGs, sncRNAs and SNPs were all linked to fertility, which was further confirmed by the modelling and feature selection approaches. Correlations between the quantitative features (CpGs and sncRNAs) best represented on the first dimension of MFA did exist within a single dataset but not between CpG and sncRNA datasets. Although correlations between these two types of epigenetic processes might perhaps be expected [42], the result we obtained was not consistent with the direct regulation of sncRNA expression by DNA methylation. However, the peculiar transcriptional status of sperm cells should be taken into account when interpreting this result. Indeed, sperm cells are transcriptionally inactive, and most of the sncRNAs that accumulate during spermatogenesis are actually piRNAs [8,43]. The sperm RNA content is then drastically modified during the transit of sperm through the epididymis, where rRFs, miRNAs and tRFs are gained. These sncRNAs gained during post-testicular maturation are not transcribed by the spermatozoa but transferred through epididymosomes. Their expression is therefore independent of the sperm DNA methylome. Furthermore, some studies have suggested that sncRNAs gained through the epididymis are important to the first stages of embryogenesis, while piRNAs are mainly important for fertility during spermatogenesis stages [44,45]. Here, we selected scnRNAs linked to fertility, but because we did not observe any major changes to semen parameters, we might speculate that some of the molecular events related to subfertility arose after spermatogenesis, involving sncRNAs acquired during epididymal transit rather than piRNAs transcribed during earlier stages. This hypothesis is in line with the observation that most of the scnRNAs identified as being related to fertility during MFA belonged to the miRNA and rRF families.

### The different models select non-redundant features

It has to be pointed out that the whole dataset was used in order to identify the predictive features and no independent dataset was available in this study to further evaluate the model performances. This aspect could lead to a phenomenon called “data leakage” which can result in an overestimation of the true prediction value of a model [46]. However, as mentioned in the previous section, sncRNAs and CpG are both linked to fertility without being correlated to each other, meaning that they bring non-redundant information on bull fertility. These elements can make us speculate that data integration will allow to improve the prediction quality of the models. Nevertheless, in order to confirm these results, another large-scale independent cohort will be needed.

An interesting aspect is that only a few features were selected in common by the different models, and their nature also differed markedly as a function of the method applied. Importantly, Logistic Lasso is an additive method, while the other methods do not make any assumptions about the relationships between features and can deal with complex non-linear interaction patterns. Furthermore, the importance of features in Logistic Lasso is assessed individually for each feature, a property shared with genome-wide associations studies (GWAS) that usually investigate the genetic association between individual SNPs and a given phenotype. Of note, only a few SNPs have so far been identified by GWAS as belonging to male fertility quantitative trait loci (QTLs) [5]. This could be explained by the fact that male fertility is a complex trait with relatively low heritability. Rather than a major gene, several SNPs in combination, each explaining a small part of the phenotypic variance, might then be involved in male subfertility (at least in normospermic cases), limiting the identification of QTLs when using classical GWAS methods. Similarly, because of its reduced ability to select relevant combinations of SNPs, Logistic Lasso may preferentially select sncRNAs and CpGs that could potentially exert greater individual influence on fertility than SNPs. This behaviour might therefore explain the huge proportion of selected sncRNAs and CpGs (242 out of 250) with respect to SNPs. The reason why Gradient Boosting and classic Random Forest also selected more sncRNAs and CpGs than SNPs is probably different. Indeed, unlike Logistic Lasso, these two methods are not purely additive and can select features with possible interactions, although they are both biased toward the selection of quantitative rather than qualitative features [32]. We therefore switched to the Cforest method, which is a Random Forest approach that can account for this bias; this produced the selection of each type of -omics data at relatively balanced proportions. Finally, the Neural Network approach, which is described as dealing with complex interactions among features, selected the highest proportion of SNPs. Based on this result, it can be hypothesized that while CpGs and scnRNAs individually contribute to fertility, a defined combination of SNPs displaying a complex correlation structure that is best accounted for by Neural Networks, was necessary to reach maximum predictive accuracy in this bull cohort.

### Which method to use in order to select phenotype linked features in mixed omics data?

This study was conducted in an integrative context, mixing data from different origins and distributions which affected the performance of the five methods investigated to different degrees. As mentioned above, classic Random Forest and Gradient Boosting were both biased regarding the distribution and nature of the data; this did not directly influence model performance but clearly affected feature selection. Of the three remaining methods, Logistic Lasso is not described as displaying such a selection bias, but it evaluates the contribution of features one by one without taking account of non-additive interactions. This property might be a drawback because in -omics integrative studies the different types of data may possibly interact in a non-additive way. The two remaining methods, Cforest and Neural Network, rely on different mathematical principles but share two properties: (i) they are not sensitive to data origin or distribution, and (ii) they both allow complex interactions between features without any *a priori*. They are therefore both well suited for prediction and feature selection in integrative analyses of–omics data. However, Cforest is easier to handle than Neural Networks; there are very few hyper parameters to optimize and the recommended parameters are relevant. On the other hand, although an artificial neuron is a simple mathematical object, Neural Networks are very complex with a large number of hyper parameters to optimize and numerous possible architectures (autoencoder, convolutional etc.). Their successful application requires both background knowledge and computing resources. This is precisely why Cforest is probably best suited for biologists who wish to build predictive models and perform feature selection from–omics data.

### Epigenetic and genetic features target different functions

Strikingly, genes involved in embryonic development were overrepresented among the putative target genes of selected miRNAs. Although it can be argued that using a software for the *in silico* identification of miRNA target genes might lead to false positives, it is noteworthy that some miRNAs identified during our study, such as miR-100 and miR-29a (which both ranked at the top of the selected miRNA features) have well established functions in embryonic development [47,48]. In addition, miR-339a, miR-449a, mir-1246 and miR-21-5p, all identified during this study, have also been highlighted as being differentially expressed between high and low fertility bulls [49,50], suggesting their relevancy to bull fertility. Finally, the selected rRF features included 18S, 12S, 28S, 16S and 5.8S subspecies. rRFs have long been regarded as degradation products of rRNAs without any biological significance. As rRFs are acquired by sperm during transit through the epididymis, they might be of potential importance to embryonic development. Moreover, they have been found to interact with the AGO proteins, suggesting a role in gene regulation in the same way as miRNAs [51]. Due to a lack of knowledge regarding the role of rRFs in fertility, this hypothesis remains speculative. It should be mentioned that one study reported differential expression for some rRFs between two groups of patients with contrasted IVF outcomes [52].

A cluster of terms related to homeobox genes was significantly enriched among the CpG features selected. This cluster included *PBX1*, *BARHL1*, *MKX*, *LHX3*, *ALX4*, *ZFHX4*, *HOXB1*, *TLX3* and *HMX1* homeobox genes that are involved in different stages of embryonic and foetal development [53–61], but it also contained two genes with a CXXC-type zinc finger motif (*CXXC1* and *CXXC5)*, and three transcription factors (*NFIX*, *TEAD2* and *ELF2)* that all play an important role in embryonic development [62–66]. Another cluster of interest concerned fibronectin, which is a glycoprotein involved in cell adhesion to the extracellular matrix. It is located at the head of spermatozoa and facilitates the interaction between spermatozoa and the oocyte [40]. To date, the proteins included in this cluster have not been reported to mediate gamete interactions, and contain a fibronectin type III domain which is involved in cell-to-cell interactions.

Interestingly, the epigenetic mechanisms target embryonic development genes. Subtle changes to the expression of developmentally important genes by an altered DNA methylation profile of the paternal genome and/or a change to the sperm sncRNA content may potentially affect the normal course of development and result in increased embryo losses, ultimately influencing male fertility without dramatic consequences. By contrast, the genetic transmission of developmentally unfavourable alleles may have more severe impacts on gestation outcomes and lead to more extreme phenotypes that were not represented in the present cohort.

## Conclusions

Using four different methodologies (Logistic Lasso, Random Forest/Cforest, Gradient Boosting and Neural Networks), predictive features of the bull fertility status have been identified and belong to the CpG, sncRNA.and SNP datasets. These features were non-redundant, highlighting the potential utility of data integration for bull fertility prediction. However, to confirm these first results, a larger independent cohort is now needed. The subset of selected features was unique to each method; this could be related to the specific behaviours of the methods towards feature distribution and correlation structure. A common characteristic shared by all the methods was that the epigenetic features which were selected (both CpG DNA methylation and sncRNA expression) pointed to embryonic development as the principal process potentially dysregulated in our cohort. As a general conclusion, this study highlighted several markers of interest related to bull fertility among a wide panel of DNA methylation markers, sncRNAs and SNPs. Tracking these markers on a routine basis may allow to predict the fertility potential of bulls. The CpGs identified in this study were included in a newly developed DNA methylation microarray, which could allow routine quality controls of bull semen at a reasonable cost, in order to identify subfertile bulls, improve the management of reproduction and reduce the economic losses related to unsuccessful AI.

## Supporting information

S1 File This file contains: S1–S9 Figs. (PDF)

S1 Table This table lists the semen samples, information on their origin and processing, and the filed fertility corresponding to the bulls. (XLSX)

S2 Table This table lists the standardized methylation values for each bull in the cohort for the CpGs that were pre-selected by Random Forest. This table also contains the gene annotations, and the method with which the optimal features have been identified.
(XLSX)

S3 Table This table lists the chromosomal locations of the SNPs that have been pre-selected by Random Forest, as well as the associated genotypes for each individual. This table also contains the gene annotations, and the method with which the optimal features have been identified.
(XLSX)

S4 Table This table lists the standardized and batch corrected sncRNA values for each bull in the cohort for the sncRNAs that were pre-selected by Random Forest. This table also contains the gene annotations, and the method with which the optimal features have been identified.
(XLSX)

S5 Table This table lists the names of the 777 features that maximise the AUC in the Neural Networks, Cforest and Logistic Lasso analyses that were used for the functional annotation. (XLSX)
