# Supplementary material for: Multi-omics data integration for the identification of biomarkers for bull fertility
Source: PLoS One. 2024 Feb 23;19(2):e0298623. doi: 10.1371/journal.pone.0298623 (PMC10890740; doi:10.1371/journal.pone.0298623)
Supplement: S1 File — (PDF) [file pone.0298623.s001.pdf]

Multi-omics data integration for the identification of biomarkers for bull fertility.

Valentin Costes[1,2,3,4], Eli Sellem[1,2,3], Sylvain Marthey[4,5], Chris Hoze[3,4], Aurélie Bonnet[1,2,3], Laurent Schibler[3], Hélène Kiefer[1,2] and Florence Jaffrezic[4*]

[1]Université Paris-Saclay, UVSQ, INRAE, BREED, 78350 Jouy-en-Josas, France.

[2]Ecole Nationale Vétérinaire d'Alfort, BREED, 94700, Maisons-Alfort, France.

[3]R&D Department, ALLICE, 149 rue de Bercy, 75012, Paris, France.

[4]Université Paris-Saclay, AgroParisTech, INRAE, GABI, 78350 Jouy-en-Josas, France.

[5]INRAE, MaIAGE, Université Paris-Saclay, 78350 Jouy-en-Josas, France.

*Corresponding author: valentin.costes@eliance.fr

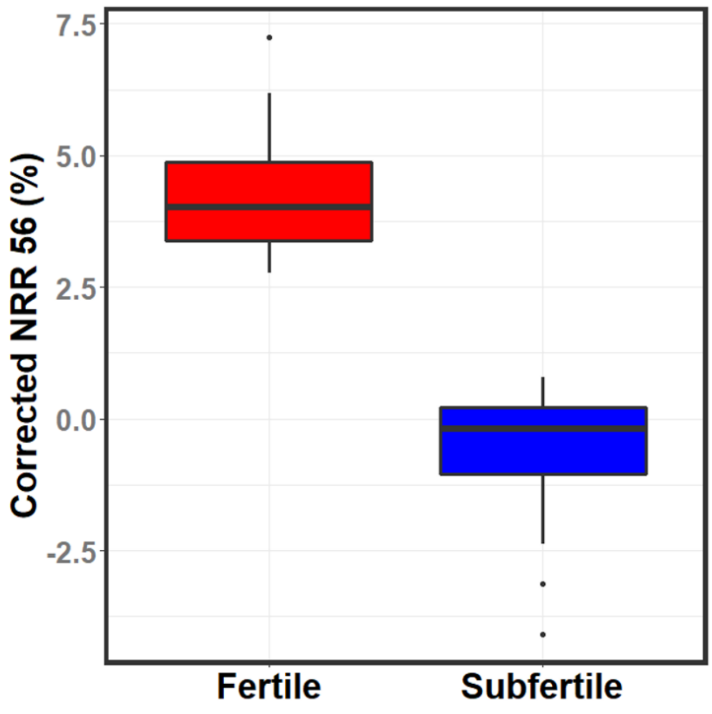

Figure S1: Corrected non return rates at 56 days (NNR 56) in fertile (red) and subfertile (blue) bulls.

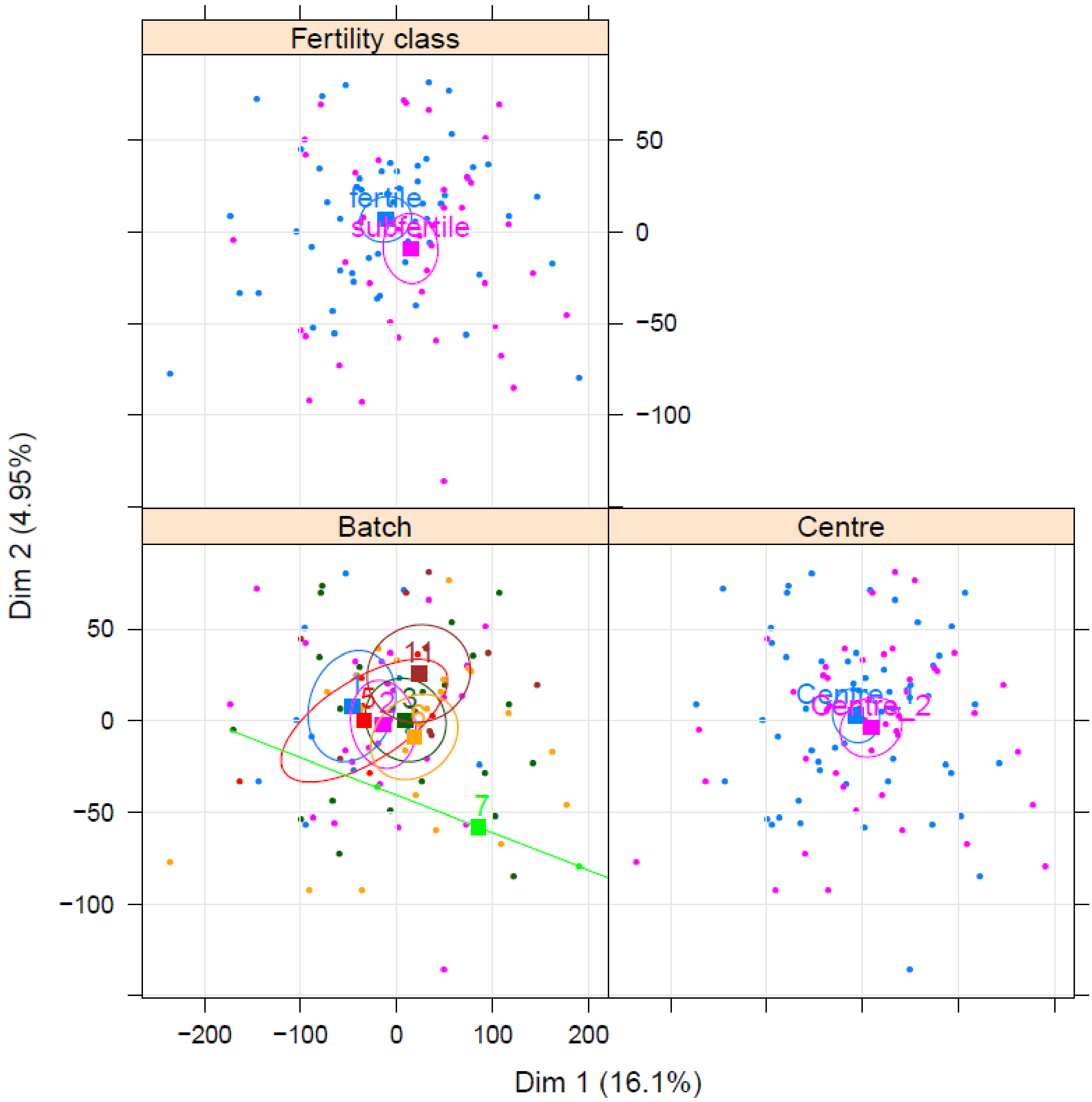

Figure S2: A PCA was run on the 40,000 CpGs remaining after the pre-filtering step and the two first dimensions are represented. The same PCA was illustrated by the fertility class (upper panel), the batch effect (lower left panel) and the semen collection centre (lower right panel). The methylation values at these CpGs were not influenced by the batches and the semen collection centres, and were therefore not corrected for these effects.

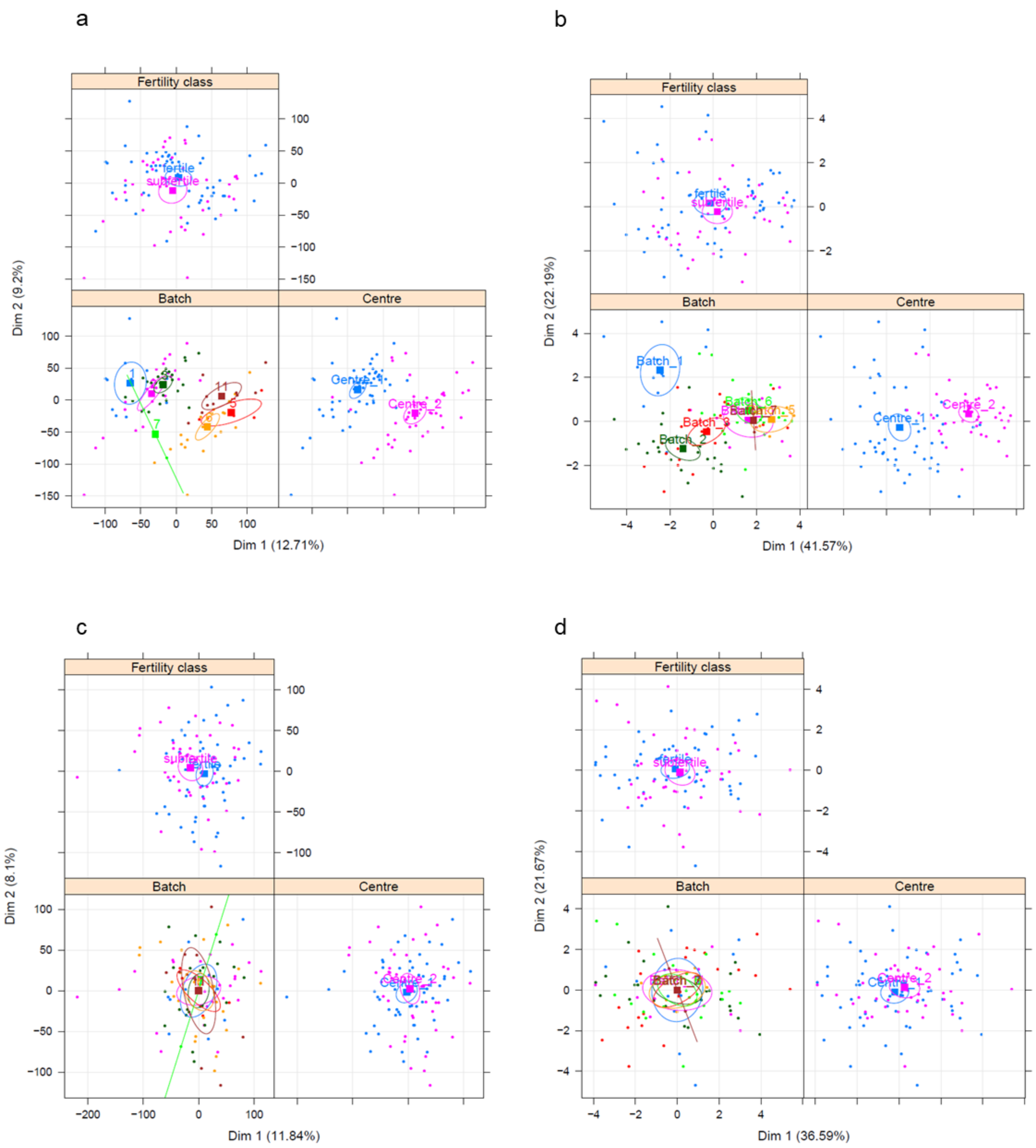
a
b
c
d
Fertility class
Batch
Centre
fertile
subfertile
Centre_1
Centre_2
Batch_1
Batch_2
Batch_3
Batch_5
Batch_6
Batch_7
Dim 1 (12.71%)
Dim 2 (9.2%)
Dim 1 (41.57%)
Dim 2 (22.19%)
Dim 1 (11.84%)
Dim 2 (8.1%)
Dim 1 (36.59%)
Dim 2 (21.67%)

Figure S3 (previous page): A PCA was run on 24,172 sncRNAs (a) and 11 semen parameters (SPs) (b) without correction. The two first dimensions are represented and three different factors were used as illustrating variables: the fertility class, the experimental batch and the semen collection centre. The experimental batch and the semen collection centre have both a huge effect on sncRNAs and SPs. After correction for the batch effect, a PCA was run on the scnRNAs (c) and SPs (d) corrected data. The corrected data are no longer biased according to the batch nor the centre.

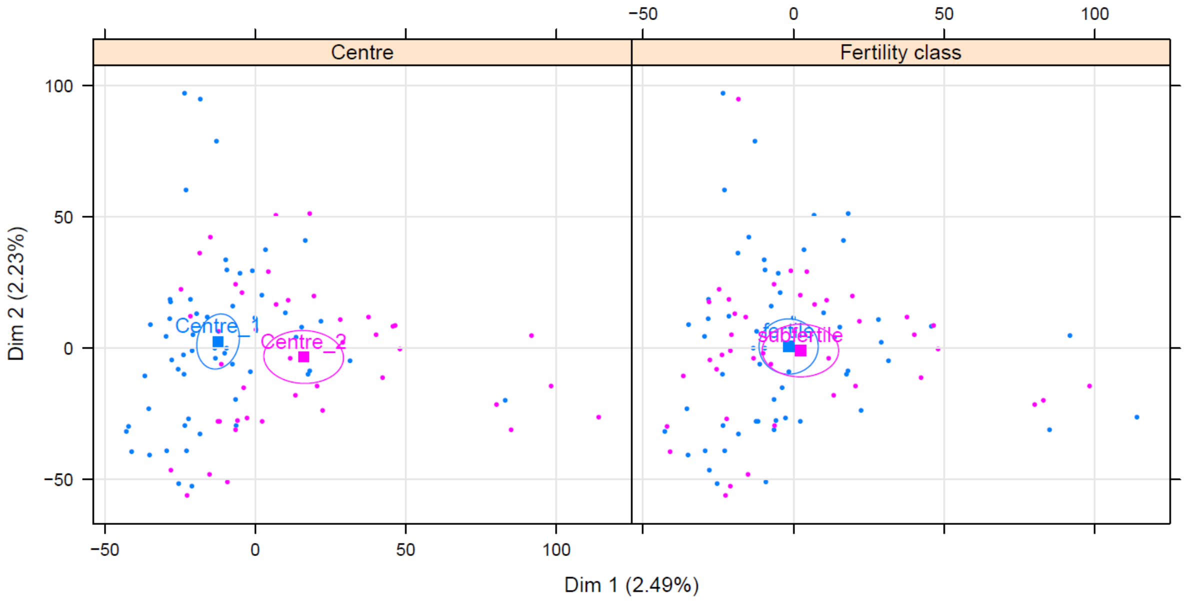

Figure S4: A PCA was run on the 38,853 SNPs remaining after the pre-filtering step and the two first dimensions are represented. The same PCA was illustrated by the semen collection centre (left panel) and the fertility class (right panel).

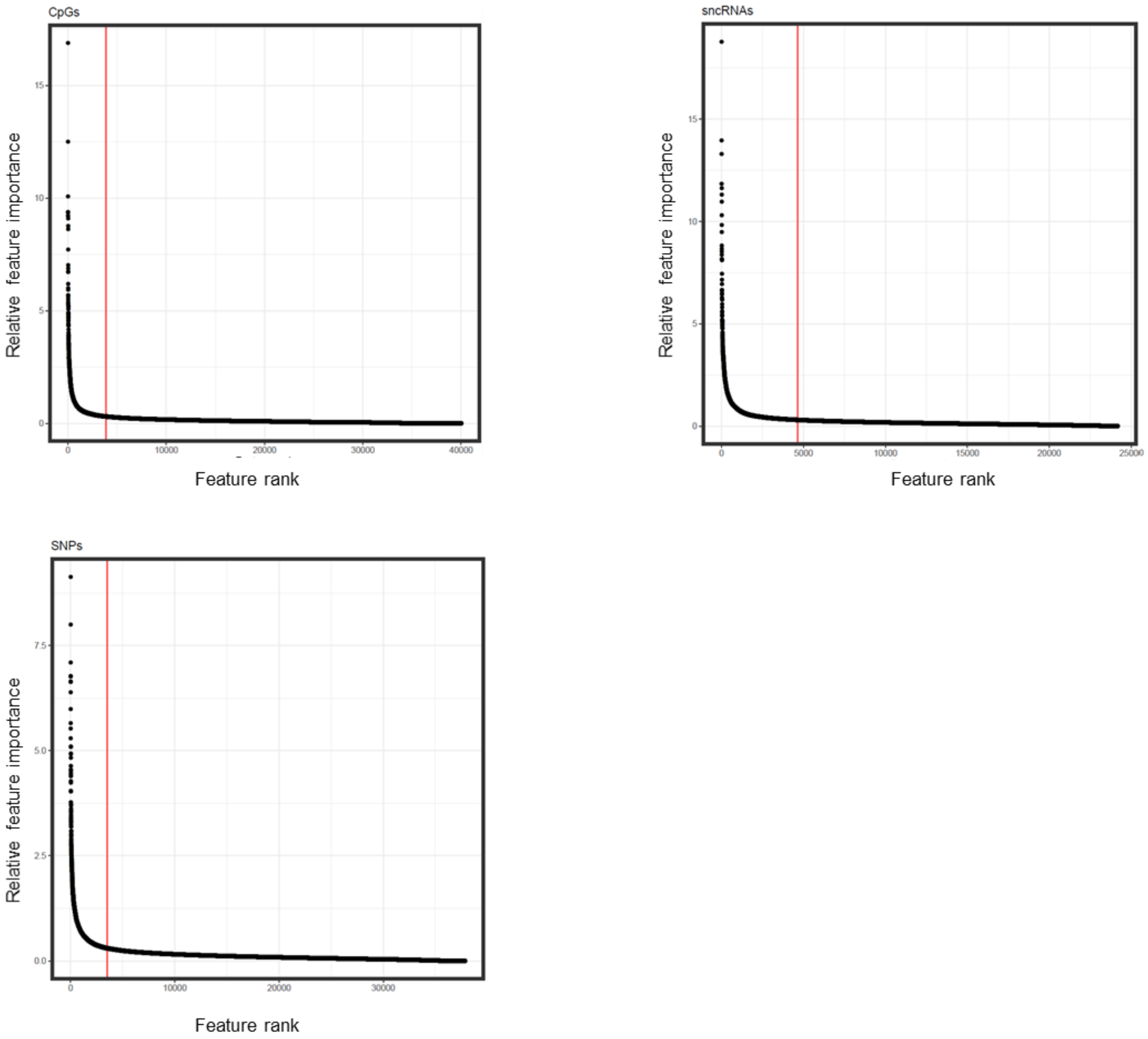

Figure S5: For each type of data, the relative feature importance of the model was plotted against the feature rank. The red vertical line indicates the threshold fixed for the pre-selection by Random Forest, where each feature on the left of this curve was selected.

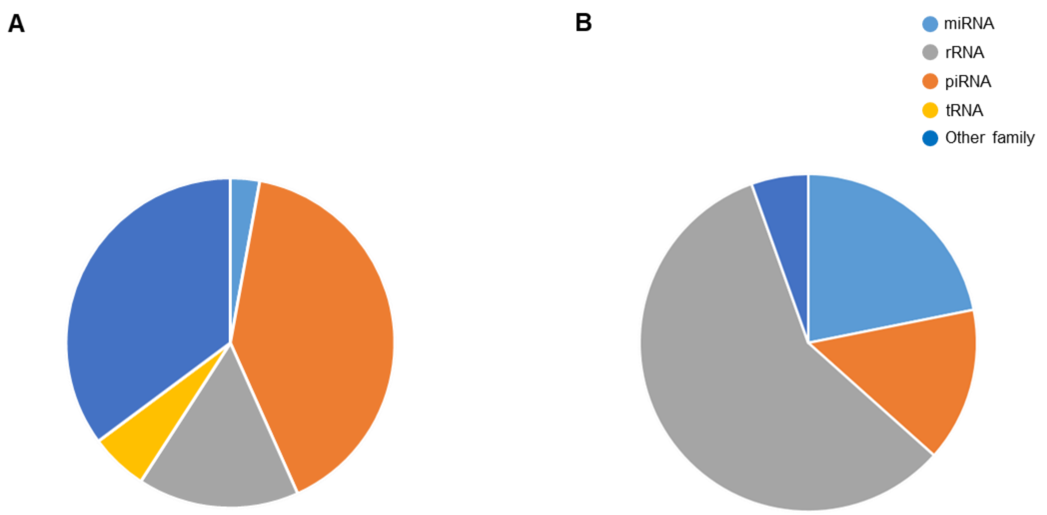

Figure S6: Pie charts showing the proportion of the different classes of sncRNAs in the background (A) and in clusters 1 and 2 that were highlighted by the MFA (Figure 2).

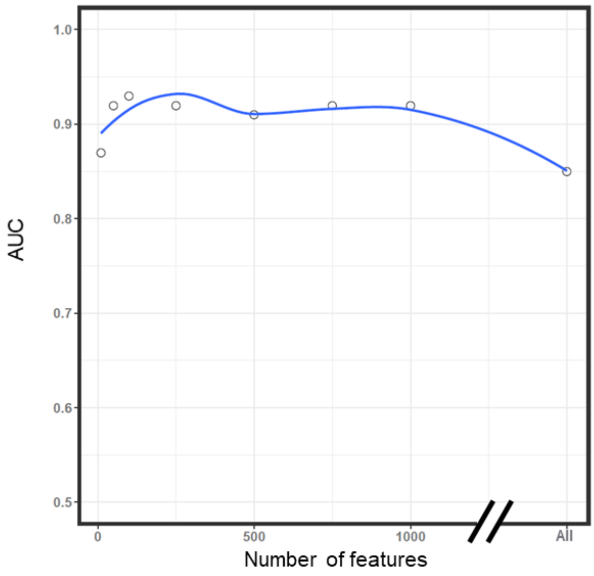

	Number of optimal features	AUC
Cforest	100	0.92

Figure S7: One model was constructed using the Cforest method with the 12,006 features and features were classified depending on their importance. Then, models were constructed with the top 1000, 750, 500, 250, 100, 50 and 10 features. Using this information, the figure on the left hand indicates the AUC on the y-axis and the number of features used during model construction on the x-axis. Each dot represents the actual AUC value obtained for each model. A tendency curve was drawn using the geom_smooth function of the ggplot2 package with default parameters. The table on the right hand shows the optimal number of features and the associated AUC value obtained for the Cforest method, based on the actual AUC values and not on the tendency curve.

A

EASE score 1.68

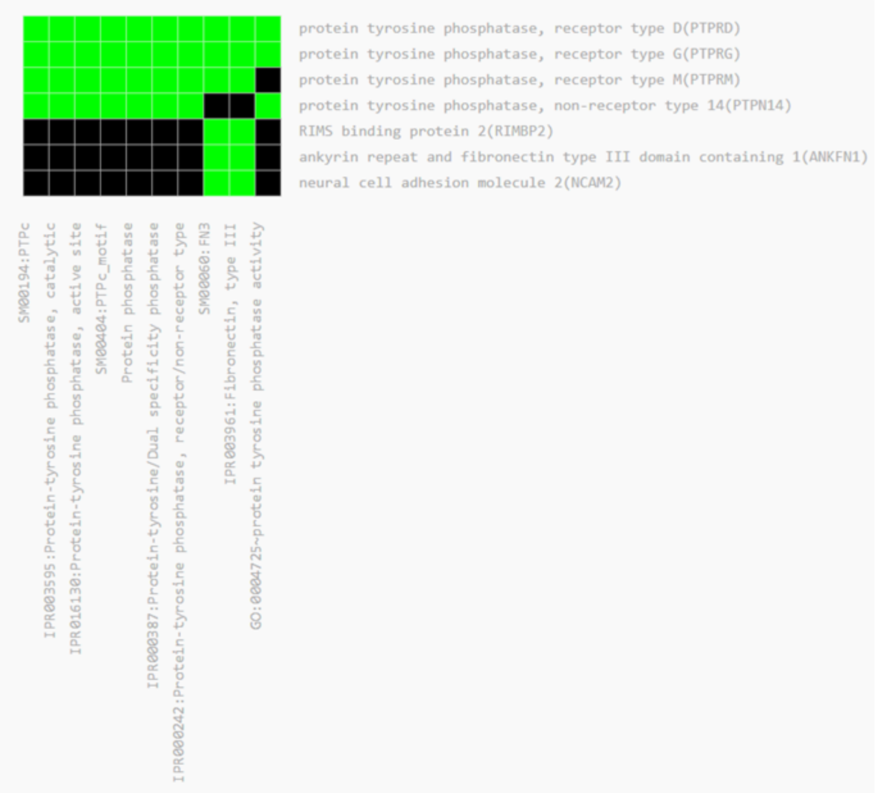

B

EASE score 1.45

EASE score 1.45

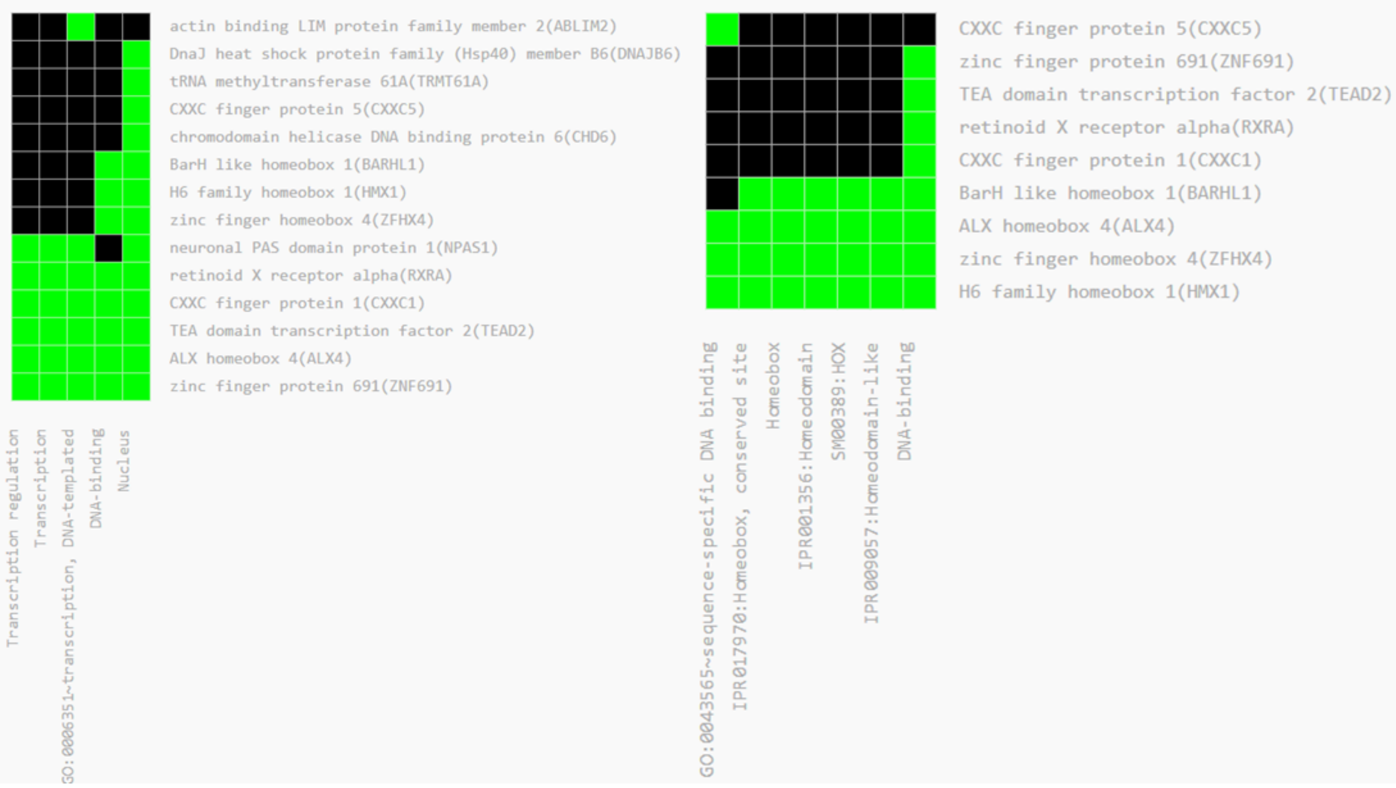

Figure S8 (previous page): The genes containing SNP and CpG features selected by Neural Networks (A) and Logistic Lasso (B) were submitted to an enrichment analysis using DAVID. Three clusters of terms were significantly enriched (EASE score above 1.3).

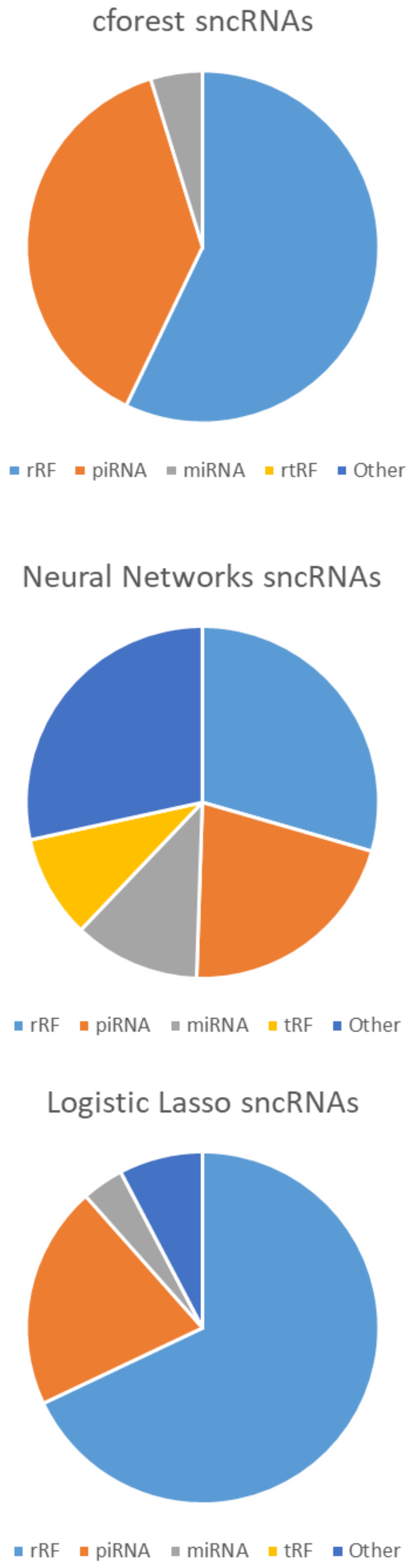

Supplementary Figure 9: Distribution of the different sncRNA families among the sncRNA features identified by each method individually.
